# Macrophage-Mediated Melanoma Reduction after HP-NAP Treatment in a Zebrafish Xenograft Model

**DOI:** 10.3390/ijms23031644

**Published:** 2022-01-31

**Authors:** Gaia Codolo, Nicola Facchinello, Nicole Papa, Ambra Bertocco, Sara Coletta, Clara Benna, Luigi Dall’Olmo, Simone Mocellin, Natascia Tiso, Marina de Bernard

**Affiliations:** 1 Department of Biology, University of Padova, 35131 Padova, Italy; gaia.codolo@unipd.it (G.C.); nicole.papa@iov.veneto.it (N.P.); sara.coletta.1@studenti.unipd.it (S.C.); 2 Department of Molecular Medicine, University of Padova, 35121 Padova, Italy; nicola.facchinello@unipd.it; 3 Soft-Tissue, Peritoneum and Melanoma Surgical Oncology Unit, Veneto Institute of Oncology IOV-IRCCS, Via Gattamelata 64, 35128 Padova, Italy; luigi.dallolmo@unipd.it (L.D.); simone.mocellin@unipd.it (S.M.); 4 Department of Biomedical Sciences, University of Padova, 35131 Padova, Italy; ambra.bertocco@phd.unipd.it; 5 Department of Surgery, Oncology and Gastroenterology (DISCOG), University of Padova, 35124 Padova, Italy; clara.benna@unipd.it

**Keywords:** HP-NAP, melanoma, zebrafish, macrophages

## Abstract

The *Helicobacter pylori* Neutrophil Activating Protein (HP-NAP) is endowed with immunomodulatory properties that make it a potential candidate for anticancer therapeutic applications. By activating cytotoxic Th1 responses, HP-NAP inhibits the growth of bladder cancer and enhances the anti-tumor activity of oncolytic viruses in the treatment of metastatic breast cancer and neuroendocrine tumors. The possibility that HP-NAP exerts its anti-tumor effect also by modulating the activity of innate immune cells has not yet been explored. Taking advantage of the zebrafish model, we examined the therapeutic efficacy of HP-NAP against metastatic human melanoma, limiting the observational window to 9 days post-fertilization, well before the maturation of the adaptive immunity. Human melanoma cells were xenotransplanted into zebrafish embryos and tracked in the presence or absence of HP-NAP. The behavior and phenotype of macrophages and the impact of their drug-induced depletion were analyzed exploiting macrophage-expressed transgenes. HP-NAP administration efficiently inhibited tumor growth and metastasis and this was accompanied by strong recruitment of macrophages with a pro-inflammatory profile at the tumor site. The depletion of macrophages almost completely abrogated the ability of HP-NAP to counteract tumor growth. Our findings highlight the pivotal role of activated macrophages in counteracting melanoma growth and support the notion that HP-NAP might become a new biological therapeutic agent for the treatment of metastatic melanomas.

## 1. Introduction

Melanoma is a cancer that develops from the uncontrolled proliferation of melanocytes, the pigment-producing cells. Mostly found on the skin, it can also occur on mucosal surfaces, the uveal tract and the leptomeninges. Historically considered as a rare cancer, in the last 50 years melanoma incidence has risen faster than almost any other cancer [1,2] and while it still represents less than 5% of all cutaneous malignancies, it is the most lethal form of skin cancer [3], which tends to spread beyond its originating site. Surgery is no longer effective in treating metastatic melanoma and the long-term prognosis is dismal, despite combined immune checkpoint inhibitors with targeted therapy drugs (for cancers with BRAF gene changes) and/or with radiation are able to extend life expectancy by some years [3].

Immune checkpoints are negative modulators of the host immune response frequently expressed by cancer cells and their inhibition leads to the amplification of anti-tumor T-cell responses, thus increasing the chance of the latter to kill the malignant cells. However, the tumor microenvironment is a complex system in which tumor cells dictate the profile of recruited immune cells. Consequently, T regulatory cells, myeloid-derived suppressor cells (MDSC) and macrophages with a pro-tumoral profile populate the tumor stroma, and altogether may limit the antitumor immunity, thus contributing to the failure of the therapy [4,5]. Notably, recruited macrophages with a pro-tumoral profile overwhelm pro-inflammatory macrophages in all stages of melanoma [6], and macrophage infiltration has been found to directly correlate with melanoma thickness and poor prognosis [7,8]. Considering the evidence of the pro-tumorigenic role for macrophages in melanoma, and the fact that innate pro-tumorigenic states are highly dynamic and can be selectively reverted, investigating the impact of skewed macrophages towards the pro-inflammatory phenotype on the clearance of melanoma tumor cells is of high translational relevance.

The Neutrophil-Activating Protein of *Helicobacter pylori* (HP-NAP) is a small dodecameric protein that acts as a major proinflammatory factor [9]. It is a chemoattractant for and activator of monocytes and immature dendritic cells (DCs), which are stimulated to mature, differentiate and release TNF-α, IL-6 and IL-12 [10]. The latter cytokine is the main responsible for the profile shifting of antigen-activated T cells from a T helper (Th) 2 toward a Th1 cytotoxic phenotype [10].Various studies demonstrated the anti-tumor potential of HP-NAP due to its activity as a modulator of the adaptive immune response [11,12,13], but the possibility that HP-NAP might counteract tumor growth due to the modulation of mononuclear cells, regardless of the participation of the adaptive immunity, remained unexplored.

The zebrafish model is an effective tool for studying tumor biology and immune system interactions [14]. The innate immune system of zebrafish is quite similar to that of vertebrates, and the adaptive immunity matures completely just 2–3 weeks after fertilization, thus providing a time window to investigate only the innate immune response, regardless of the adaptive branch [15]. Moreover, taking advantage of engineered reporter lines, the unique transparency of the zebrafish embryos allows visualizing the interactions of multiple cell types by imaging.

Here we set up a zebrafish model of melanoma xenograft, based on the injection of labeled human tumor cells into embryos and the assessment of tumor behavior in response to HP-NAP. By this assay, we demonstrated that HP-NAP efficiently inhibits melanoma growth and metastasis, and we propose that macrophages play a pivotal role in this process. The ability of HP-NAP in reverting the macrophage profile from pro-tumoral to anti-tumoral is likely to be the key element for the clearance process.

## 2. Results

### 2.1. HP-NAP Administration Counteracts Melanoma Growth and Metastasis In Vivo

**Figure 1:**
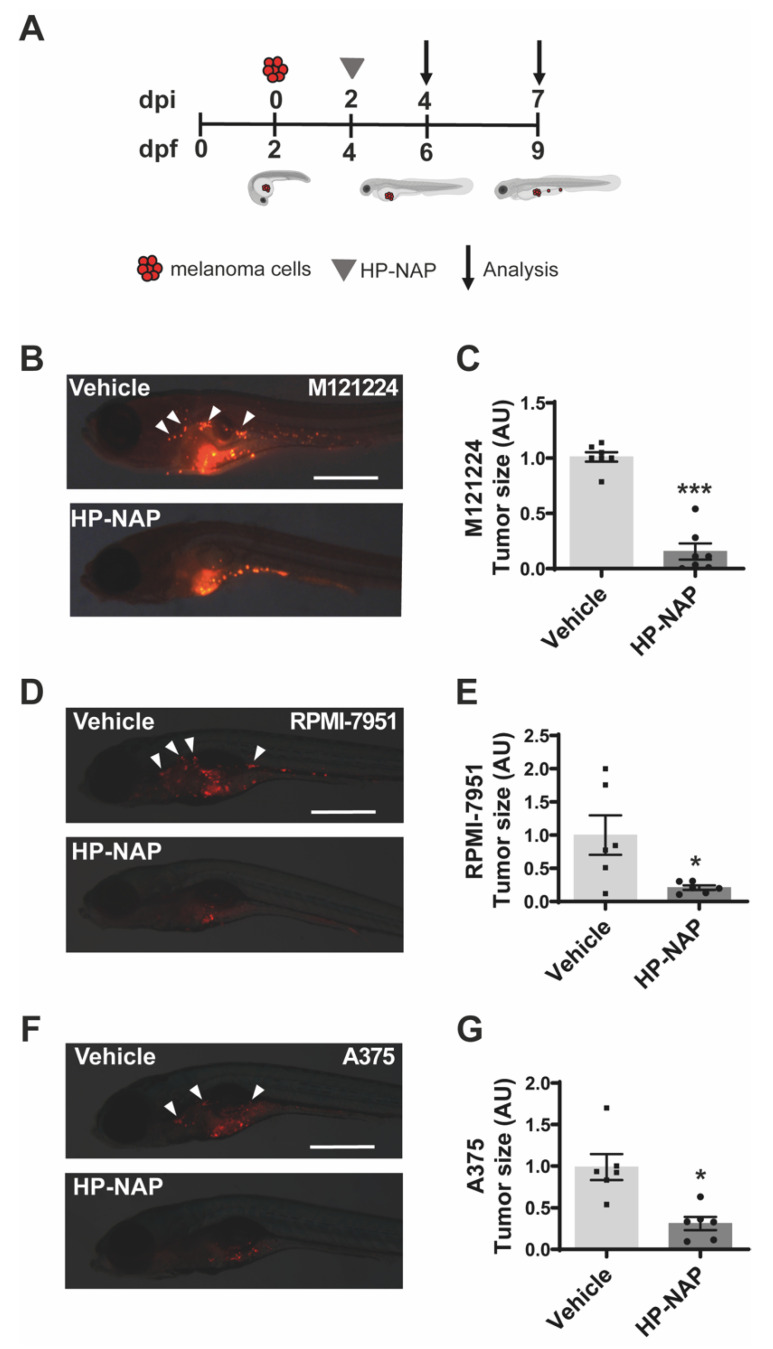
Effect of HP-NAP on melanoma growth in zebrafish model. (**A**) Scheme of the experimental design. M121224, RPMI-7951 or A375 human melanoma cells were stained with Vybrant^TM^ DiI, xenotransplanted into the yolk of transparent zebrafish larvae at 2 days post-fertilization (2 dpf)/0 days post-injection (0 dpi). At 4 dpf/2 dpi, embryos were injected with HP-NAP or saline (vehicle) as a negative control, and were observed at 9 dpf/7 dpi. (**B**,**D**,**F**) Representative fluorescence stereoscope images of embryos injected with M121224, RPMI-7951 and A375 cells respectively and treated with HP-NAP or vehicle. Arrowheads indicate metastases. Scale bar: 500 µm. (**C**,**E**,**G**) Scatter plots show the quantification of the tumor size (AU: Arbitrary Unit) at 9 dpf/7 dpi. Values are expressed as mean ± SEM and analyzed by Student’s *t* test; *, *p* < 0.05 and ***, *p* < 0.001; *n* = 7 for M121224 cells and *n* = 6 for RPMI-7951 and A375 cells (for each vehicle and HP-NAP treatment).

With the aim to assess the capacity of HP-NAP to affect melanoma growth, we took advantage of a xenograft model of human melanoma in zebrafish [16,17]. We injected three different human melanoma cell lines (M121224, RPMI-7951 and A375) stained with Vybrant^TM^ DiI, into the zebrafish yolk at 2 days post-fertilization (2 dpf), as depicted in Figure 1A. Two days after injection (2 dpi, 4 dpf), zebrafish larvae were injected with vehicle or HP-NAP into the yolk and tumor growth, and metastasis formation were monitored up to 7 dpi (9 dpf). Injected melanoma cells formed a large mass in the control group, and the detachment of metastatic cells from the primary tumor was clearly valuable. HP-NAP-treated fishes also developed the tumor, but the size of the mass was significantly lower (*p* < 0.001) (Figure 1B–G). Considering that M121224 cells gave rise to larger tumor masses in fish than the other two cell lines, we decided to adopt this melanoma cell line for deciphering the mechanism(s) underpinning the anti-tumor activity of HP-NAP.

**Figure 2:**
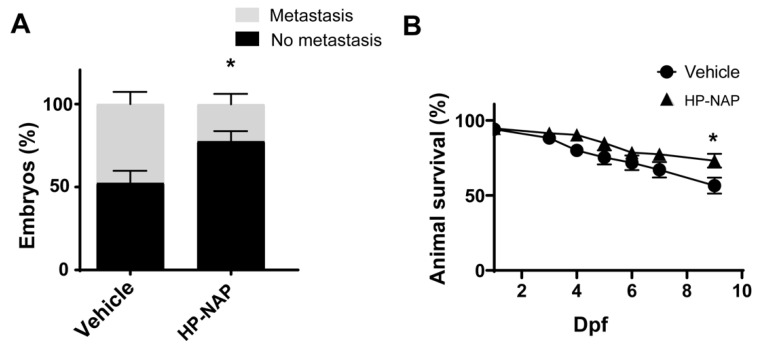
Effect of HP-NAP on melanoma metastasis and survival in zebrafish model. Fishes were treated as reported in Figure 1A. (**A**) The number of larvae with or without metastasis was counted at 9 dpf/7 dpi and expressed as percentage of the total embryos’ population. Values are expressed as mean ± SEM and analyzed by two-way ANOVA; *, *p* < 0.05; *n* = 150 fishes for each treatment. (**B**) Kaplan-Meier survival curves of animals treated with HP-NAP vs. vehicle; *n* = 50 fishes for each treatment. The Log Rank test was used for statistical analysis. *, *p* < 0.05.

Interestingly, in HP-NAP-treated larvae, most of the cells remained confined in the yolk area at the site of injection (77.5% ± 6.23 vs. 52.39 ± 7.38 of vehicle-treated individuals), resulting in a significant reduction of the number of larvae with metastasis (22.5% ± 6.23) with respect to control fishes (47.61% ± 7.38) (Figure 2A). 

Interestingly, the percentage of animals alive at 7 dpi was higher in the group of HP-NAP-treated animals than in control animals (72.12% vs. 56.47%, *p* < 0.05) (Figure 2B). In accordance, xenotransplanted embryos stained with the vital dye acridine orange showed rare fluorescent spots in HP-NAP animals, both at 4 and 7 dpi. Conversely, the number of dead cells per embryo was relevant in the control group (Supplementary Figure S1).

### 2.2. The Anti-Tumor Effect of HP-NAP Does Not Rely on a Direct Action on Melanoma Cells

**Figure 3:**
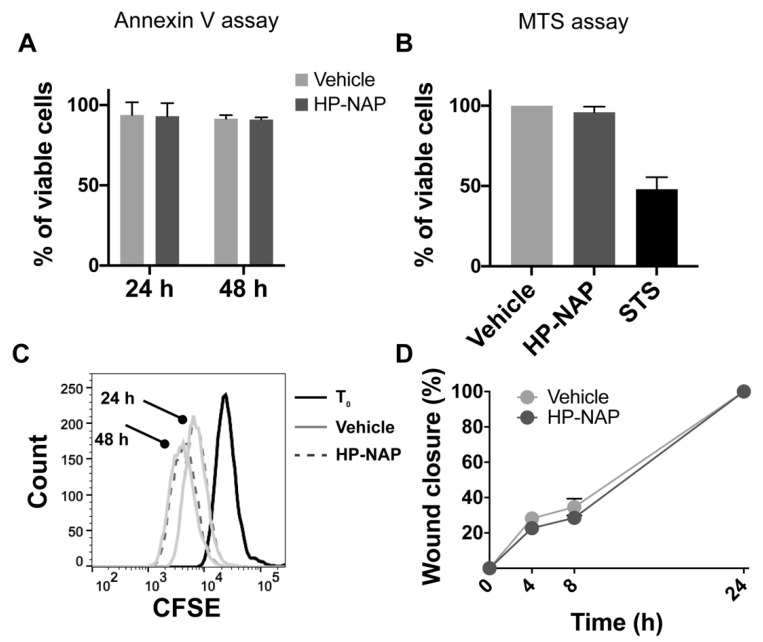
HP-NAP has no direct effect on melanoma cells. (**A**) M121224 cells were treated with HP-NAP or saline (vehicle) and after 24 and 48 h, apoptosis was evaluated by annexin V staining. (**B**) M121224 cells were treated with HP-NAP, saline (vehicle) or staurosporine (STS) as positive control; cytotoxicity was evaluated by MTS assay. Values are reported as percentage of viable cells ± SEM of three independent experiments. (**C**) M121224 cells were labeled with CSFE and treated with HP-NAP or saline (vehicle) for 24 and 48 h. Cells were harvested, washed, resuspended in saline and analyzed by flow cytometry. Representative histograms from two independent experiments are reported. (**D**) Cell migration was evaluated after a 24 h incubation with HP-NAP in serum-free RPMI (supplemented with 0.1% BSA). Cells incubated in medium with the same volume of saline were considered as control (vehicle). 0.1 × 10^6^ M121224 cells were seeded in a 24-well culture dish. Scratches were created once cells reached confluence. At time zero and after 4, 8 and 24 h, wound closures were photographed under a microscope. Migration rate was expressed as percentage of wound closure (0%: T_0_ after wound; 100%: completely repaired).

To investigate whether HP-NAP triggered apoptosis in melanoma cells, we performed an Annexin-V assay on M121224 exposed for 24 and 48 h to HP-NAP, but we did not reveal any effect on cell viability, with respect to control cells (Figure 3A). An MTS assay was carried out to definitively rule out any cytotoxic effect (Figure 3B). Then, we evaluated the effect of HP-NAP treatment on the proliferation of cancer cells, and to this purpose, a CFSE-based proliferation assay was performed. We observed that after 24 and 48 h, the proliferation rate of M121224 cells was comparable between cells exposed to HP-NAP and those untreated (Figure 3C). On the same line, HP-NAP did not impact the migration of cancer cells, as revealed by the wound-healing assay (Figure 3D).

Collectively, these data demonstrated that the anti-tumor activity of HP-NAP did not rely on a direct effect on tumor cells.

### 2.3. HP-NAP Promotes the Recruitment of Macrophages at the Tumor Site

**Figure 4:**
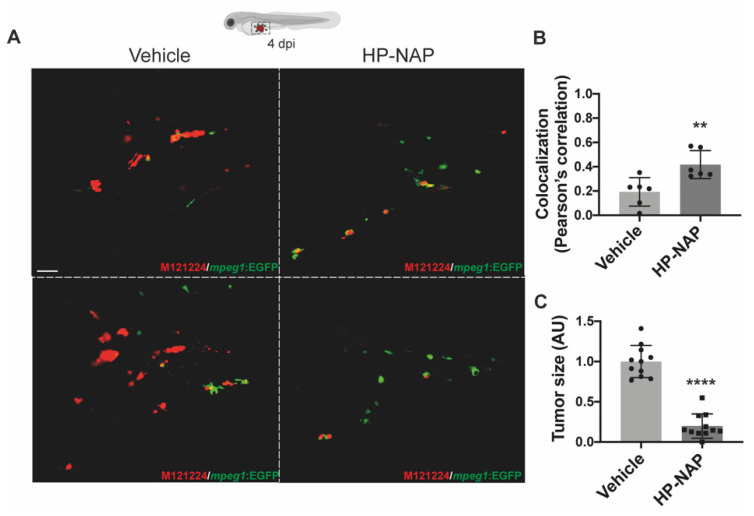
HP-NAP treatment favors the interaction between tumor cells and macrophages and early affects tumor size. Tg(mpeg1:EGFP)gl22 zebrafish embryos were xenotransplanted with M121224 melanoma cells (red), injected or not with HP-NAP at 2 dpi and observed at 4 dpi. (**A**) Representative 2D projections of confocal single plane images of the yolk-sac region of embryos at 4 dpi. Magnification: 40×. Scale bar: 20 µm. (**B**) Scatter plots show the colocalization (Pearson’s correlation) between green (macrophages) and red (tumor cells) signals in vehicle- and HP-NAP-injected fishes. At least 5 Regions of Interest (ROIs) per sample of 3 independent experiments were analyzed. Values are shown as mean ± SEM and analyzed by Student’s *t* test; **, *p* < 0.01; *n* = 6 for each condition. (**C**) Scatter plots show the quantification of the tumor size (AU: Arbitrary Unit) at 4 dpi. Values are expressed as mean ± SEM and analyzed by Student’s *t* test; ****, *p* < 0.0001; *n* = 11 for each condition, from 3 independent experiments.

After excluding any direct cytotoxic or cytostatic effect of HP-NAP on melanoma cells, we explored the possibility that HP-NAP injection could generate a tumor suppressive microenvironment. Since the observational window of larvae was limited to 9 dpf, well before the maturation of the adaptive immunity, and considering that macrophages accumulate in melanoma [6], we first addressed whether the injection of HP-NAP could induce a more robust accumulation of these cells around the tumor, with respect to what occurred in untreated animals. To this aim, we took advantage of the zebrafish transgenic line Tg(mpeg1:eGFP)gl22, which has macrophages fluorescently labelled [18]. M121224 cells were injected into the fish, and after 2 d larvae were treated or not treated with HP-NAP. 48 h later (4 dpi), animals observed by confocal microscopy revealed that not only was the number of macrophages recruited at the site of injection higher in animals receiving HP-NAP, but a major proportion of them were in close contact with melanoma cells, which notably were few and less agglomerated than in control fishes (Figure 4A and Supplementary Video S1). Quantification of the localization of melanoma cells and macrophages and of the tumor size confirmed the microscopy observation (Figure 4B,C).

### 2.4. HP-NAP Modulates the Macrophages Polarization

**Figure 5:**
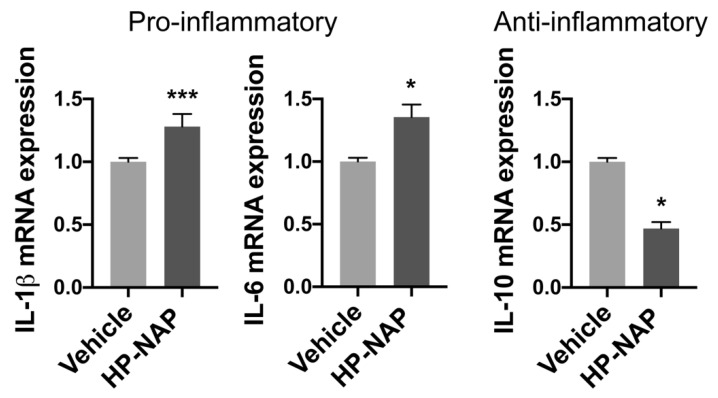
HP-NAP administration in zebrafish promotes the acquisition of a pro-inflammatory profile by macrophages. Tg(mpeg1:EGFP)gl22 zebrafish embryos were xenotransplanted with M121224 melanoma cells and treated with HP-NAP or saline (vehicle) at 2 dpi. After 24 h (3 dpi) macrophages were FACS-sorted and the expression of genes was evaluated by qRT-PCR. Data were normalized to the housekeeping gene 18S. The expression of each gene in macrophages isolated from HP-NAP-treated fishes was relative to that in macrophages isolated from vehicle animals, taken as reference and set as 1. Data are expressed as the mean ± SEM of 2 independent experiments and analyzed by Student’s *t* test; *, *p* < 0.05; ***, *p* < 0.001.

Afterward, we investigated the possibility that HP-NAP might also inhibit tumor progression by shifting the phenotype of recruited macrophages toward a pro-inflammatory, anti-tumor profile. Hence, we sorted macrophages from Tg(mpeg1:eGFP)gl22 larvae, treated or not treated with HP-NAP, 4 d after the transplantation of melanoma cells (Supplementary Figure S2) and we determined the expression pattern of pro-inflammatory (il-1β and il-6) and immunoregulatory (il-10) cytokines. According to our prediction, macrophages isolated from HP-NAP animals expressed significantly more il-1β and il-6 than those from control animals (Figure 5 left panels), while they showed the opposite behavior in terms of il-10 expression (Figure 5 right panel).

### 2.5. Macrophages Are Essential for the Anti-Tumor Activity of HP-NAP

**Figure 6:**
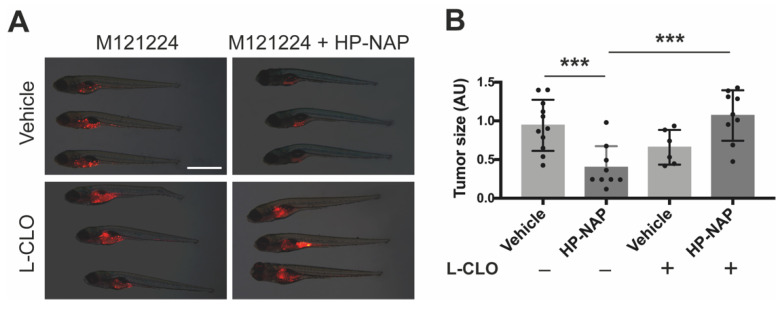
The anti-tumor activity of HP-NAP is strictly dependent on macrophages: effect of macrophage depletion by liposome-encapsulated clodronate (L-CLO). Tg(mpeg1:EGFP)gl22 zebrafish embryos were xenotransplanted with M121224 melanoma cells (red) and treated with empty liposomes + saline (vehicle), or empty liposomes + HP-NAP, L-CLO + saline (L-CLO) or L-CLO + HP-NAP at 2 dpi and observed at 4 dpi. (**A**) Representative fluorescence stereoscope images of the total tumor mass in fishes at 4 dpi. Scale bar: 1 mm. (**B**) Scatter plots show the quantification of the total tumor size (AU: Arbitrary Unit) at 4 dpi. Values are expressed as mean ± SEM and analyzed by one-way ANOVA; ***, *p* < 0.001, HP-NAP (*n* = 9) vs. vehicle (*n* = 11) and L-CLO + HP-NAP (*n* = 9) vs. HP-NAP (*n* = 11).

**Figure 7:**
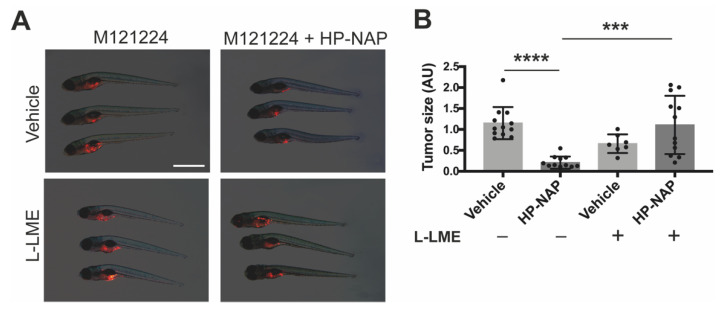
The anti-tumor activity of HP-NAP is strictly dependent on macrophages: effect of macrophage depletion by L-Leucyl L-Leucine Methyl Esther (L-LME). Tg(mpeg1:EGFP)gl22 zebrafish embryos were xenotransplanted with M121224 melanoma cells (red) and treated with saline (vehicle), or L-leucine methyl ester (L-LME), L-LME + HP-NAP or HP-NAP at 2 dpi and observed at 4 dpi. (**A**) Representative fluorescence stereoscope images of the total tumor mass in fishes at 4 dpi. Scale bar, 1 mm. (**B**) Scatter plots show the quantification of the total tumor size (AU: Arbitrary Unit) at 4 dpi. Values are expressed as mean ± SEM and analyzed by one-way ANOVA; ****, *p* < 0.0001, HP-NAP vs. vehicle (*n* = 12 for each condition); ***, *p* < 0.01: L-LME + HP-NAP (*n* = 12) vs. HP-NAP (*n* = 12).

To further substantiate the role of macrophages in HP-NAP-induced tumor clearance, we repeated the above experimental set up in Tg(mpeg1:eGFP)gl22 fishes depleted for most macrophage population through the administration of Liposome-Clodronate (L-CLO), which targets macrophages regardless of their embryonic origin [19]. The massive decrease of macrophages (Supplementary Figure S3A) jeopardized the anti-tumoral activity of HP-NAP, as testified by the melanoma size that was the same as that of control fishes (Figure 6A,B). We reached the same conclusion when we depleted macrophages by administrating to the fishes L-LME (Supplementary Figure S3B), a lysosomotropic compound known to be selectively toxic to macrophages, NK cells, cytotoxic T lymphocytes [20] and skin mast cells [21] (Figure 7A,B).

## 3. Discussion

Solid tumors generate a detectable immune response that, unfortunately, instead of blocking tumor progression, frequently gets hijacked and contributes to tumor growth. The immune contexture dictated by tumors excludes cytotoxic T cells or favors an exhausted T cell phenotype via a plethora of immune-evasive signals, frequently involving innate immune cells [22].

The fact that tumor-infiltrating macrophages are critical orchestrators of a microenvironment that supports tumor progression is now an accepted paradigm. On the other hand, macrophages have a remarkable plasticity, and if properly trained, can mediate powerful antitumor actions by eliminating malignant cells. Pro-inflammatory macrophages directly mediate cytotoxicity to kill tumor cells. They release tumor killing molecules such as TNF-α, reactive oxygen radicals and nitric oxide, or actively take up cancer cells by phagocytosis [23]. Moreover, pro-inflammatory macrophages activate the killing potential of natural killer (NK) cells [24].

Despite several preclinical and clinical studies have shown that shifting the phenotype of tumor-infiltrating macrophages toward a pro-inflammatory, anti-tumor profile inhibits tumor progression [25,26], strategies that revitalize innate immune cells restoring their ability to recognize and destroy tumor cells are underrepresented in current immuno-oncology therapies.

The presence of macrophages in primary lesions has been revealed in melanomas [7] and elevated numbers of macrophages within a melanoma are markedly associated with poor prognosis [27], supporting the notion that melanoma macrophages are polarized toward an immune suppressive pro-tumor profile. Moreover, they are considered to play a central role in negatively affecting melanoma response to immune checkpoint inhibitors [28]. Consequently, approaches aimed at depleting or reprogramming pro-tumor macrophages were proposed to circumvent the failure of therapies based on immune checkpoint blockade.

In 2006, we revealed that the *Helicobacter pylori* protein HP-NAP acts as a Toll-like receptor-2 agonist, and that it triggers the release of pro-inflammatory/Th1 polarizing cytokines, namely IL-6, TNF-α and IL-12 by innate immune cells [10]. Several studies investigating the anti-tumor potential of HP-NAP in in-vivo tumor models followed our pioneer study [11,12,13], but all of them ascribed the efficacy of HP-NAP in counteracting tumor growth to the activity on the adaptive immune response.

In the present study, by taking advantage of a whole-vertebrate model of human melanoma, we aimed to investigate the capacity of HP-NAP to revert the immune suppressive profile of macrophages educated by cancer cells, and to assess the role of the switched phagocytes in tumor regression. In accordance with previous evidence [29], we confirmed that in-vivo, HP-NAP is chemotactic for macrophages. In addition, we revealed that the protein administration enabled phagocytic cells to acquire a pro-inflammatory/anti-tumor profile, which was crucial for hampering melanoma growth and metastasis. By performing our analysis at the zebrafish larval stage, we could evaluate the anticancer therapeutic activity of HP-NAP net of any adaptive immune response, and this not only allowed us to finely dissect the effects of the protein on the innate immunity components, but also highlighted the potential and feasibility of a HP-NAP-based cancer therapy, in case of a compromised activity of lymphocytes. It is worthy to mention that HP-NAP treatment also led to an increase of the overall survival of animals.

To conclude, there are two main novelties of our study: the first is finding that reprogramming tumor macrophages is a strategy to successfully counteract melanoma growth; and the second is shedding light upon the ability of the bacterial protein HP-NAP in counteracting tumor growth, even in the absence of the acquired/specific branch of the immune system. Our evidence supports the notion that HP-NAP might become a new biological therapeutic agent for the treatment of metastatic melanoma, as such, or in combination with blockers of immune checkpoints.

## 4. Materials and Methods

### 4.1. Ethic Statement

Investigation has been conducted following the ethical standards, the Declaration of Helsinki and national and international guidelines (Directive 2010/63/EU), and with permission for animal experimentation of the Ethics Committee of the University of Padova and the Italian Ministry of Health (Authorization number 407/2015-PR).

### 4.2. Danio Rerio (Zebrafish) Handling

The following zebrafish lines were used for these studies: wild type Tuebingen and the transgenic line Tg(mpeg1:eGFP)gl22, in which the macrophage population is Green Fluorescent.

Zebrafish were maintained in a temperature-controlled (28.5 °C) environment in a 12:12 light/dark cycle, and fed as described by Kimmel et al. (1995) [30]. For anesthesia or euthanasia of zebrafish embryos and larvae, tricaine (E10521, Sigma-Aldrich, St. Louis, MO, USA) was added to the fish water at 0.16 mg/mL or 0.3 mg/mL, respectively.

### 4.3. Human Melanoma Cell Lines

Human melanoma M121224 (University Research Priority Program–Melanoma Biobank Zurich), RPMI-7951 (HTB-66, ATCC, Manassas, VA, USA) and A375 (CRL-1619, ATCC) cell lines were used for the experiments. M121224 cells were grown in RPMI 1640 medium (Euroclone, Milan, Italy), supplemented with 10% Fetal Bovine Serum (FBS, Euroclone), 100 U/mL Penicillin, 100 µg/mL Streptomycin (Sigma-Aldrich), 10 mM HEPES buffer (Euroclone). RPMI-7951 and A375 were kept in EMEM and DMEM media (Euroclone), respectively, and both supplemented with 10% FBS, Penicillin, Streptomycin and 10 mM HEPES.

All cell lines were maintained at 37 °C in humidified atmosphere containing 5% CO_2_ atmosphere, and routinely tested for mycoplasma contamination.

### 4.4. Annexin V Assay

M121224 cells were treated or not with 20 µg/mL HP-NAP. After 24 and 48 h cells were harvested and incubated with the phosphatidylserine-binding protein annexin V FITC-conjugated/propidium iodide (PI) (Bender MedSystem, Wien, Austria) according to the manufacturer’s instructions. Viability was measured by flow cytometry (BD LSR Fortessa XL20, BD Bioscience). Results were analyzed with FlowJoTM v10.6.1 software, and expressed as percentage of annexin V/PI-negative events [31].

### 4.5. MTS Assay

Cell cytotoxicity was evaluated using the tetrazolium reduction (MTS) assay. Cells were seeded into 96-well plates at a density of 5 × 10^3^ cells/well, and allowed to grow in RPMI 1640 medium (supplemented as described before) for 24 h. Growth medium was then replaced with phenol red- and FBS-free medium and treated or not with 20 µg/mL HP-NAP or 4 µM staurosporine as positive control. After 24 h, 10% CellTiter 96^®^ AQUEOUS One solution (Promega, Milano, Italy) was added to each well, as indicated by the supplier. After a 4 h incubation at 37 °C, absorbance at 490 nm was measured using an Infinite^®^ 200 PRO 96-well plate reader.

### 4.6. Proliferation Assay

M121224 cells were labeled with 2 µM carboxyfluorescein diacetate succinimidyl ester (CSFE; ThermoFisher Scientific, Waltham, MA, USA), following the manufacturer’s instructions. Labelled cells (0.2 × 10^5^) were plated in a 24-well culture plate, and stimulated or not with 20 µg/mL HP-NAP. Proliferation was assessed after 24 and 48 h by flow cytometry (BD LSR Fortessa XL20, BD Bioscience, San Jose, CA), and analyzed with FlowJoTM v10.6.1 software.

### 4.7. Migration Assay

Cell migration was evaluated using the wound healing assay. 0.1 × 10^6^ M121224 cells were plated in a 24-well culture dish, and upon reaching confluence, a scratch was made through each well using a sterile pipette tip. Debris were removed by gentle washing with medium, and 20 μg/mL HP-NAP was added. Cells were monitored under the microscope (magnification, 10×) at time 0 and after 4, 8 and 24 h (at 37 °C in 5% CO_2_). Images of cells were captured at the same position before and after the treatment to document the repair process. Images were individually analyzed using ImageJ (v.2.0), and the percent of wound closure was calculated.

### 4.8. Xenotransplant

For xenotransplantation, embryos were mechanically dechorionated at 2 d post-fertilization (dpf), anesthetized (Tricaine 0.16 mg/mL), and placed along plastic lanes immersed in 2% methylcellulose/PBS. Melanoma cells were stained for 20 min at 37 °C with Vybrant^TM^ DiI Cell-Labeling Solution (5 μg/mL, Molecular Probes, Eugene, OR, USA), which contains a lipophilic membrane stain that diffuses laterally to label the entire cell. Stained cells were resuspended in 10 μL PBS (or selected medium for each condition) at a density of 0.2 × 10^6^ cells/µL, loaded in a glass capillary needle and microinjected into the yolk (about 50 cells/embryo), using a WPI PicoPump apparatus. At 1 d post-injection (dpi), zebrafish that displayed cells in the blood vessels were discarded.

Xenotransplanted embryos were then grown at 33 °C, monitored daily and documented at 4 and 7 dpi (experimental endpoint). At each observation time point, died animals were discarded.

Imaging was performed using a Leica M165FC dissecting microscope equipped with a Leica DFC7000T camera. Metastases were quantified and classified as (1) No metastasis: cells in place, confined to the site of injection; (2) Metastasis: cells spread from yolk to near organs (heart, swim bladder and pharynx) or in distant organs, such as brain, skeletal muscle and trunk. Moreover, the fluorescence intensities emitted from injected cells were quantified by using ImageJ (v.2.0).

### 4.9. Macrophage Depletion and HP-NAP Administration

At 2 dpi, 600 μg/mL of HP-NAP in sterile saline was injected in the zebrafish yolk (5 nL/embryo). Equal volume of saline was injected in control animals. To selectively ablate macrophages, Clodronate (L-CLO) and control (empty) liposomes (Liposoma BV) were microinjected and administrated twice, at 0 dpi (5 nL/embryo; solutions at 5 mg/mL) and 2 dpi, by resuspending Vybrant^TM^ DiI-labelled M121224 cells in L-CLO and empty liposomes.

As alternative method for macrophage depletion, the L-Leucyl L-Leucine methyl esther compound (L-LME; L1002, Sigma-Aldrich), was used. 20 mM L-LME was administered twice (0 dpi and 2 dpi) as described for L-CLO. Where required, at 2 dpi fishes were also microinjected with 600 μg/mL of HP-NAP, together with the second dose of L-CLO and L-LME.

### 4.10. Fluorescent Staining with Acridine Orange (AO)

Acridine orange (AO) staining is a nucleic acid selective metachromatic stain technique that identifies dead cells. For AO fluorescent staining, live larvae were incubated in a solution containing a final concentration of 5 mg/mL of AO (Sigma-Aldrich) in fish water at 28.5 °C for 30 min in the dark. Larvae were then washed 2–3 times in fish water and mounted in 1% low-melting agarose for confocal microscope image acquisition (TCS SP5 and Stellaris, Leica, Wetzlar, Germany). Quantification of apoptotic/necrotic AO-positive spots was performed with ImageJ (v2.0), focusing on the same intestinal area and adopting a constant signal threshold for all samples.

### 4.11. Images Acquisition and Analysis of Zebrafish Embryos

The red fluorescent tumor mass was monitored on anaesthetized embryos every day by a Leica M165FC dissecting microscope equipped with a Leica DFC7000T camera. To also detect the Tg(mpeg1:eGFP)gl22 signal, larvae were anaesthetized and mounted in 1% low melting point-agarose gel. Embryos were analyzed at high resolution with a TCS SP5 and SP8 confocal microscopes (Leica). Open-source public domain software such as ImageJ/Fiji was used for signal quantification to calculate the integrated densities in the region of interest (ROI), as previously reported [32]. In addition, we set up the threshold that best isolated positive regions from the background. All voxels with intensities lower than threshold were discarded from calculations. To compare different experiments, data were expressed in arbitrary units (AU). For all images in which the levels of fluorescence were compared, settings for laser excitation and confocal scanning detection were kept constant between groups.

### 4.12. Macrophage Sorting

Zebrafish Tg(mpeg1:eGFP)gl22 larvae treated with HP-NAP or untreated were dissociated at 3 dpi, as previously described [33]. Briefly, about 30 larvae per condition were digested in lysis solution (PBS, 0.25% trypsin phenol red free, 1 mM EDTA, pH 8.0 and 2.2 mg/mL Collagenase P (Sigma-Aldrich)) for 60 min. Digestion was stopped by adding PBS, 1 mM CaCl_2_ and 10% FBS. Dissociated cells were rinsed once in PBS and resuspended in Opti-MEM (ThermoFisher Scientific), 1% FBS and Penicillin, Streptomycin solution. Cells were filtered through a 40-μm nylon membrane. Cells were sorted using FACS Aria III sorter (BD Biosciences) with the following settings for EGFP: argon-ion Innova Laser (Coherent; 488 nm, 100 mW), 100 μM nozzle and sorting speed 500 events/s in 0-32-0 sort precision mode. Data acquisition was performed with BD FACSDiva software (BD Biosciences). GFP+ and GFP− cells were separately collected in resuspension medium and processed for subsequent analysis. The detailed gating strategy adopted is reposted in Supplementary Figure S2.

### 4.13. RNA Extraction and Quantitative Real-Time PCR (qPCR)

Total RNA from macrophages was extracted with TRIzol reagent (Thermo Fisher Scientific), according to the manufacturer’s protocol. One μg of total RNA was retrotranscribed using the High-Capacity cDNA Reverse Transcription Kit (ThermoFisher Scientific), following the manufacturer’s instructions. Retrotranscription was performed at 37 °C for 2 h, following the manufacturer’s instructions. After precipitation, 10 ng cDNA were used in qRT-PCR performed in a QuantStudio5 Real-Time PCR System (Thermo Fisher Scientific). qRT-PCR was performed in 10 µL using SYBR Green master mix (Thermo Fisher Scientific) according to the following cycle: 95 °C for 5 min, 95 °C for 15 s and 60 °C for 1 min, for 40 cycles. For each sample, data were normalized to the endogenous reference gene 18s. Sequence of primers used are listed in Table 1. Data analysis was carried out according to the ΔΔCt method, as previously described [34].

### 4.14. Statistical Analysis

Statistical analyses for zebrafish experiments were performed with GraphPad Prism v8.2. All data were based on at least two independent experiments with independent biological replicates. The Shapiro-Wilk normality test was used to confirm the normality of the data. If data passed the normality test (alpha = 0.05), then a parametric test, such as an unpaired t test or ordinary one-way ANOVA (in case of multiple comparisons), was used. If data did not pass the normality test, a non-parametric test was used (Mann-Whitney test or Kruskal-Wallis test for multiple comparisons). To correct for multiple comparisons, either Tukey’s multiple comparisons test (for ordinary one-way ANOVA) or Dunnet’s multiple comparisons test (for Kruskal-Wallis test) was used. The value of sample size (n) is given in figure legends, and for in-vivo experiments it indicates the number of animals/samples. Data are presented as mean and single points, with standard error of the mean (SEM), as indicated in figure legends. Statistical analyses for in vitro experiments were performed with GraphPad Prism v8.2. Results were expressed as the mean ± SEM. A *p* value <0.05 was considered significant.

## Figures and Tables

**Table 1:** Primes used for the amplification of zebrafish transcripts.

Gene	Sequence (5′-3′)
*rna18s*	F: GCCTGCGGCTTAATTTGACT R: ACCACCCACAGAATCGAGAAA
*il-1b*	F: GACATGCTCATGGCGAACG R: GCAAATCGTGCATTGCAAGACG
*il-6*	F: GTGAAGACACTCAGAGACG R: GTTAGACATCTTTCCGTGCTG
*il-10*	F:TCAGAGCAGGAGAGTCGAATGCA R: CGATTGGGGTTGTGGAGTGCTT

## Supplementary Materials

The following supporting information can be downloaded at: https://www.mdpi.com/article/10.3390/ijms23031644/s1.

## References

[1] Davis L.E., Shalin S.C., Tackett A.J. (2019). Current State of Melanoma Diagnosis and Treatment. Cancer Biol. Ther..

[2] Matthews N.H., Li W.-Q., Qureshi A.A., Weinstock M.A., Cho E., Ward W.H., Farma J.M. (2017). Epidemiology of Melanoma. Cutaneous Melanoma: Etiology and Therapy.

[3] Domingues B., Lopes J.M., Soares P., Pópulo H. (2018). Melanoma Treatment in Review. Immunotarg. Ther..

[4] Gentles A.J., Newman A.M., Liu C.L., Bratman S.V., Feng W., Kim D., Nair V.S., Xu Y., Khuong A., Hoang C.D. (2015). The Prognostic Landscape of Genes and Infiltrating Immune Cells across Human Cancers. Nat. Med..

[5] Cassetta L., Pollard J.W. (2018). Targeting Macrophages: Therapeutic Approaches in Cancer. Nat. Rev. Drug Discov..

[6] Falleni M., Savi F., Tosi D., Agape E., Cerri A., Moneghini L., Bulfamante G.P. (2017). M1 and M2 Macrophages’ Clinicopathological Significance in Cutaneous Melanoma. Melanoma Res..

[7] Hussein M.R. (2006). Tumour-Associated Macrophages and Melanoma Tumourigenesis: Integrating the Complexity. Int. J. Exp. Pathol..

[8] Piaggio F., Kondylis V., Pastorino F., Di Paolo D., Perri P., Cossu I., Schorn F., Marinaccio C., Murgia D., Daga A. (2016). A Novel Liposomal Clodronate Depletes Tumor-Associated Macrophages in Primary and Metastatic Melanoma: Anti-Angiogenic and Anti-Tumor Effects. J. Control. Release.

[9] Montecucco C., de Bernard M. (2003). Molecular and Cellular Mechanisms of Action of the Vacuolating Cytotoxin (VacA) and Neutrophil-Activating Protein (HP-NAP) Virulence Factors of Helicobacter Pylori. Microbes Infect..

[10] Amedei A., Cappon A., Codolo G., Cabrelle A., Polenghi A., Benagiano M., Tasca E., Azzurri A., D’Elios M.M., Del Prete G. (2006). The Neutrophil-Activating Protein of Helicobacter Pylori Promotes Th1 Immune Responses. J. Clin. Investig..

[11] Codolo G., Fassan M., Munari F., Volpe A., Bassi P., Rugge M., Pagano F., D’Elios M.M., de Bernard M. (2012). HP-NAP Inhibits the Growth of Bladder Cancer in Mice by Activating a Cytotoxic Th1 Response. Cancer Immunol. Immunother..

[12] Iankov I.D., Allen C., Federspiel M.J., Myers R.M., Peng K.W., Ingle J.N., Russell S.J., Galanis E. (2012). Expression of Immunomodulatory Neutrophil-Activating Protein of Helicobacter Pylori Enhances the Antitumor Activity of Oncolytic Measles Virus. Mol. Ther..

[13] Ramachandran M., Yu D., Wanders A., Essand M., Eriksson F. (2013). An Infection-Enhanced Oncolytic Adenovirus Secreting H. Pylori Neutrophil-Activating Protein with Therapeutic Effects on Neuroendocrine Tumors. Mol. Ther..

[14] Wyatt R.A., Trieu N.P.V., Crawford B.D. (2017). Zebrafish Xenograft: An Evolutionary Experiment in Tumour Biology. Genes.

[15] Póvoa V., Rebelo de Almeida C., Maia-Gil M., Sobral D., Domingues M., Martinez-Lopez M., de Almeida Fuzeta M., Silva C., Grosso A.R., Fior R. (2021). Innate Immune Evasion Revealed in a Colorectal Zebrafish Xenograft Model. Nat. Commun..

[16] Bootorabi F., Manouchehri H., Changizi R., Barker H., Palazzo E., Saltari A., Parikka M., Pincelli C., Aspatwar A. (2017). Zebrafish as a Model Organism for the Development of Drugs for Skin Cancer. Int. J. Mol. Sci..

[17] Kaufman C.K. (2016). Zebrafish Melanoma. Adv. Exp. Med. Biol..

[18] Travnickova J., Tran Chau V., Julien E., Mateos-Langerak J., Gonzalez C., Lelièvre E., Lutfalla G., Tavian M., Kissa K. (2015). Primitive Macrophages Control HSPC Mobilization and Definitive Haematopoiesis. Nat. Commun..

[19] Weissman B.A., Schwartz S.D., Lee D.A. (1991). Oxygen Transmissibility of Disposable Hydrogel Contact Lenses. CLAO J..

[20] Thiele D.L., Lipsky P.E. (1986). The Immunosuppressive Activity of L-Leucyl-L-Leucine Methyl Ester: Selective Ablation of Cytotoxic Lymphocytes and Monocytes. J. Immunol..

[21] Hagforsen E., Paivandy A., Lampinen M., Weström S., Calounova G., Melo F.R., Rollman O., Pejler G. (2015). Ablation of Human Skin Mast Cells in Situ by Lysosomotropic Agents. Exp. Dermatol..

[22] Gajewski T.F., Schreiber H., Fu Y.-X. (2013). Innate and Adaptive Immune Cells in the Tumor Microenvironment. Nat. Immunol..

[23] Pan Y., Yu Y., Wang X., Zhang T. (2020). Tumor-Associated Macrophages in Tumor Immunity. Front. Immunol..

[24] Bellora F., Castriconi R., Dondero A., Reggiardo G., Moretta L., Mantovani A., Moretta A., Bottino C. (2010). The Interaction of Human Natural Killer Cells with Either Unpolarized or Polarized Macrophages Results in Different Functional Outcomes. Proc. Natl. Acad. Sci. USA.

[25] Long K.B., Beatty G.L. (2013). Harnessing the Antitumor Potential of Macrophages for Cancer Immunotherapy. Oncoimmunology.

[26] Duan Z., Luo Y. (2021). Targeting Macrophages in Cancer Immunotherapy. Signal Transduct. Target. Ther..

[27] Mrad M., Imbert C., Garcia V., Rambow F., Therville N., Carpentier S., Ségui B., Levade T., Azar R., Marine J.-C. (2016). Downregulation of Sphingosine Kinase-1 Induces Protective Tumor Immunity by Promoting M1 Macrophage Response in Melanoma. Oncotarget.

[28] Ceci C., Atzori M.G., Lacal P.M., Graziani G. (2020). Targeting Tumor-Associated Macrophages to Increase the Efficacy of Immune Checkpoint Inhibitors: A Glimpse into Novel Therapeutic Approaches for Metastatic Melanoma. Cancers.

[29] D’Elios M.M., Amedei A., Cappon A., Del Prete G., de Bernard M. (2007). The Neutrophil-Activating Protein of *Helicobacter Pylori* (HP-NAP) as an Immune Modulating Agent. FEMS Immunol. Med. Microbiol..

[30] Kimmel C.B., Ballard W.W., Kimmel S.R., Ullmann B., Schilling T.F. (1995). Stages of Embryonic Development of the Zebrafish. Dev. Dyn..

[31] Casellato A., Rossi Paccani S., Barrile R., Bossi F., Ciucchi L., Codolo G., Pizza M., Aricò B., de Bernard M. (2014). The C2 Fragment from Neisseria Meningitidis Antigen NHBA Increases Endothelial Permeability by Destabilizing Adherens Junctions. Cell Microbiol..

[32] Facchinello N., Schiavone M., Vettori A., Argenton F., Tiso N. (2016). Monitoring Wnt Signaling in Zebrafish Using Fluorescent Biosensors. Methods Mol. Biol..

[33] Camillo C., Facchinello N., Villari G., Mana G., Gioelli N., Sandri C., Astone M., Tortarolo D., Clapero F., Gays D. (2021). LPHN2 Inhibits Vascular Permeability by Differential Control of Endothelial Cell Adhesion. J. Cell Biol..

[34] Pozzobon T., Facchinello N., Bossi F., Capitani N., Benagiano M., Di Benedetto G., Zennaro C., West N., Codolo G., Bernardini M. (2016). Treponema Pallidum (Syphilis) Antigen TpF1 Induces Angiogenesis through the Activation of the IL-8 Pathway. Sci. Rep..

