# Supplementary material for: Macrophage-Mediated Melanoma Reduction after HP-NAP Treatment in a Zebrafish Xenograft Model
Source: Int J Mol Sci. 2022 Jan 31;23(3):1644. doi: 10.3390/ijms23031644 (PMC8836027; doi:10.3390/ijms23031644)

Supplementary Figure S1

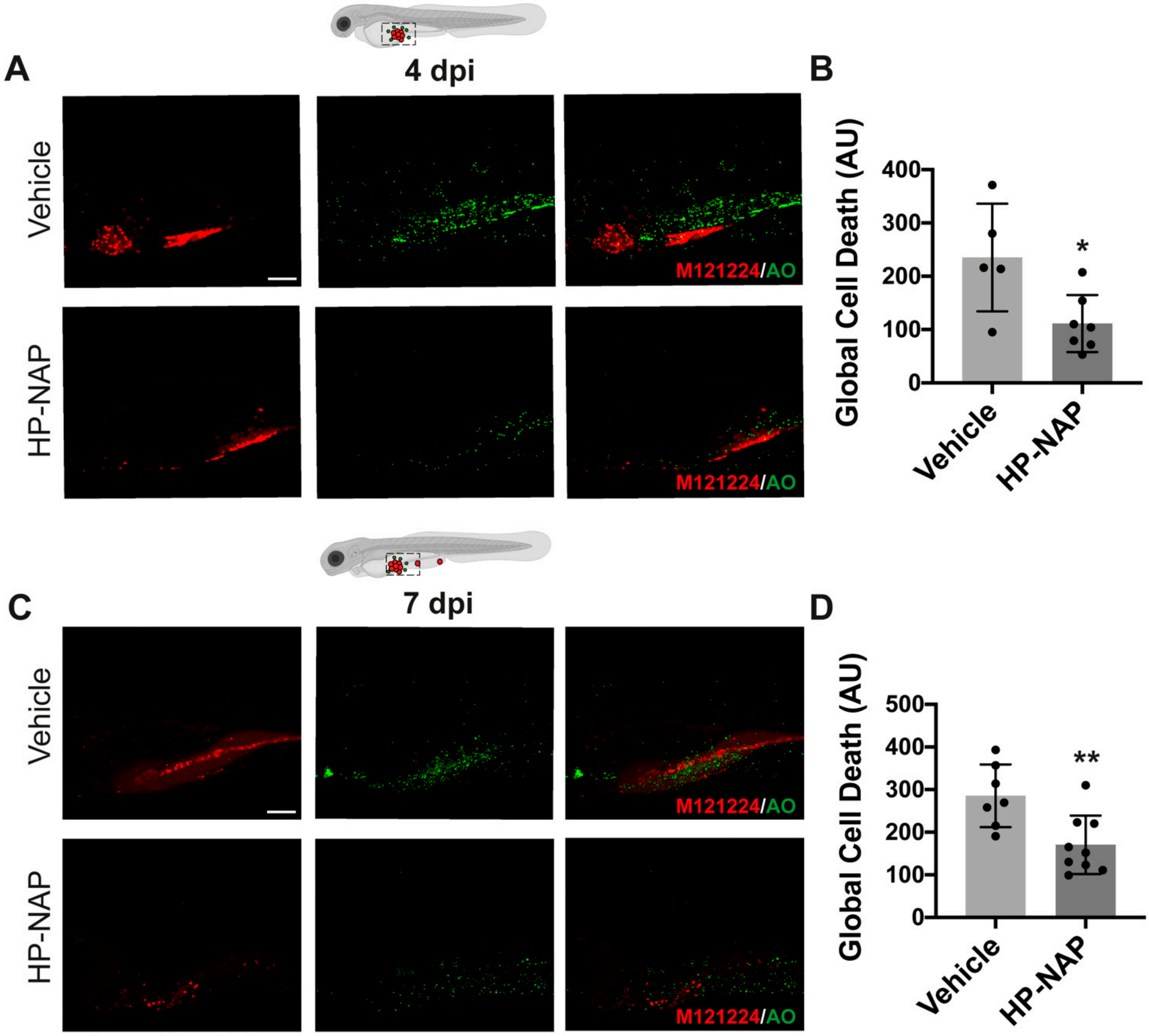

Figure S1. (**A**) Representative Z-projections of confocal images of the yolk-sac region, stained with Acridine Orange (AO, green dots), in 4 dpi larvae injected with M121224 human melanoma cells (red signals) and treated with vehicle or HP-NAP. Scale bar: 100 µm. **B.** Scatter plots show the quantification (AU) of the cell death marker AO. Values are expressed as mean ± SEM and analyzed by Student's *t* test; *, $p<0.05$; vehicle ($n=5$) vs. HP-NAP ($n=7$). **C.** Representative Z-projections of confocal images of the yolk-sac region, stained with Acridine Orange (AO, green dots), in 7 dpi larvae injected with M121224 human melanoma cells (red signals) and treated with vehicle or HP-NAP. Scale bar: 100 µm. **D.** Scatter plots show the quantification (AU) of the cell death marker AO. Values are shown as mean ± SEM and analyzed by Student's *t* test; **, $p<0.01$; HP-NAP ($n=9$) vs. vehicle ($n=7$).

Supplementary Figure S2

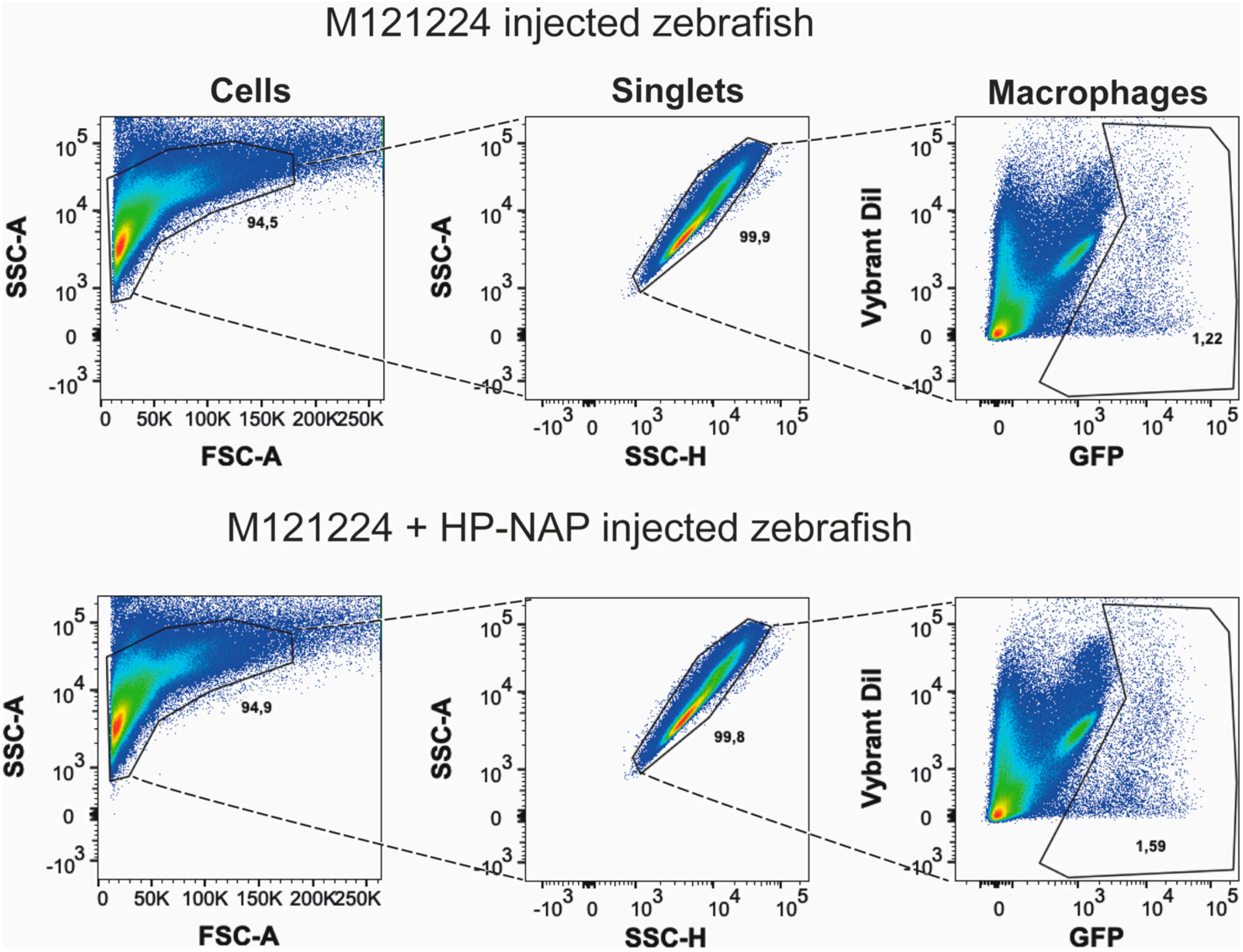

Figure S2. Representative gating strategy of macrophages sorting. Zebrafish Tg(mpeg1:eGFP)gl22 larvae, treated with HP-NAP or untreated, were dissociated at 3 dpi, digested to obtain single cell suspension and GFP^{+} macrophages were sorted using FACS Aria III sorter. Cells from M121224-injected zebrafish treated or not with HP-NAP were first gated, based on the FCS-A and SSC-A, then single cells were gated based on SSC-A and SSC-H. Macrophages were gated based on GFP expression.

Supplementary Figure S3

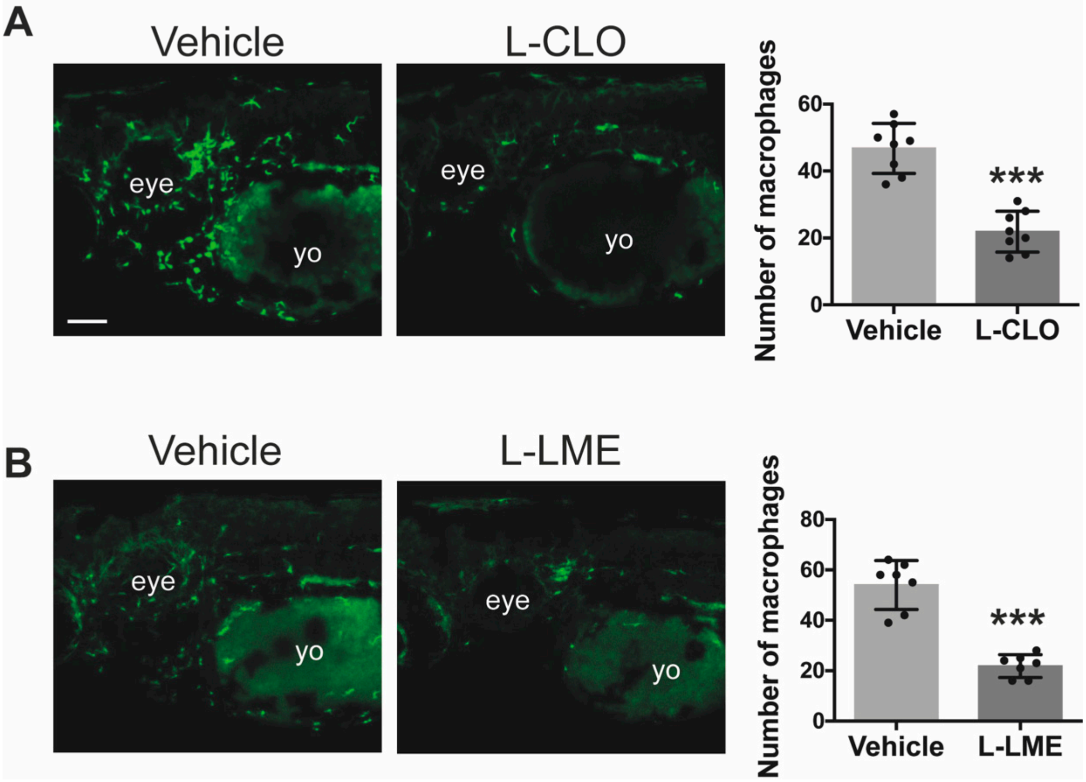

Figure S3. Depletion of macrophages with liposome-encapsulated clodronate (L-CLO) and L-Leucyl L-Leucine Methyl Esther (L-LME). (**A**) Representative Z-projections of confocal images of Tg(mpeg1:EGFP)gl22 in 3 dpf embryos (head, lateral view) transplanted with M121224 cells. Images show the decreased number of macrophages (green signals) 24 h after the administration of L-CLO compared to empty liposomes (vehicle)**.** Scale bar: 200 µm. Scatter plots show the number of macrophages in the selected area; ***, $p<0.001$; $n= 8$ for each condition. (**B**) Representative Z-projections of confocal images of embryos at 3 dpf (head, lateral view). Images show the decreased number of macrophages (green signals) 24 h after the administration of L-LME compared to vehicle**.** Scale bar: 200 µm. Scatter plots show the number of macrophages in the selected area; ***, $p<0.001$; $n=7$ for each condition. **yo**: yolk (diffused green fluorescence due to auto-fluorescence).

Supplementary Video S1: 3D reconstruction of macrophages and melanoma cells.

3D reconstruction of macrophages (green, *Tg(mpeg1:EGFP)gl22*) and melanoma cells (red, M121224) in vehicle- (**A**) and HP-NAP-treated (**B**) larvae at 4 dpi.

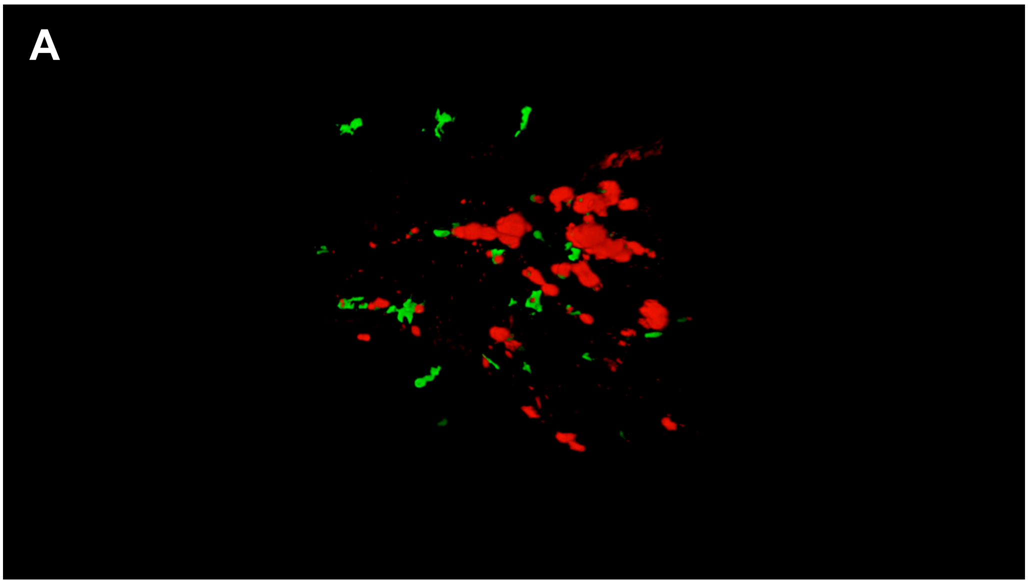

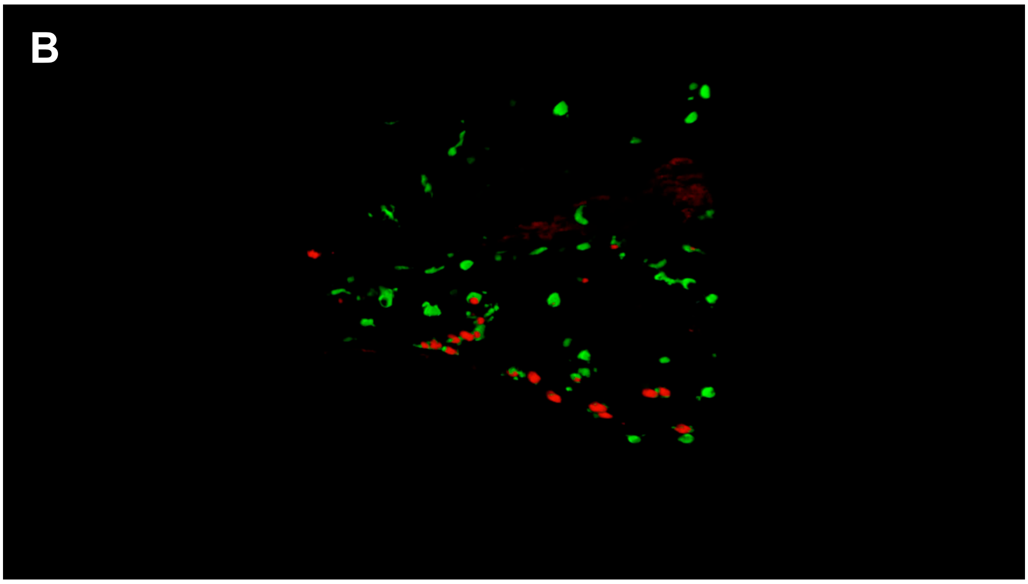

Supplement: Supplementary file 1 [file ijms-23-01644-s001.zip › ijms-1553039-supplementary.pdf]
